# Towards Ethical International Research Partnerships in Gender-Based Violence Research: Insights From Research Partners in Kenya

**DOI:** 10.1177/10778012211035798

**Published:** 2021-10-16

**Authors:** Sanne Weber, Margaret Hardiman, Wangu Kanja, Siân Thomas, Nicole Robinson-Edwards, Caroline Bradbury-Jones

**Affiliations:** 1 1724 Department of International Development, University of Birmingham, Birmingham, UK; 2 Institute for Mental Health, University of Birmingham, Birmingham, UK; 3 Wangu Kanja Foundation, Nairobi, Kenya; 4 Department of Social Work and Social Care, University of Birmingham, Birmingham, UK; 5 Institute for Global Innovation, University of Birmingham, Birmingham, UK; 6 School of Nursing, University of Birmingham, Birmingham, UK

**Keywords:** engagement, ethics, gender-based violence, participation, research methods

## Abstract

Research with survivors of gender-based violence in low- and middle-income countries is important to improve understanding of experiences of violence and the policies that can help combat it. But this research also implies risks for survivors, such as re-traumatization, safety concerns, and feelings of exploitation. These risks are magnified if research is undertaken by researchers from high-income countries, whose positionality produces power inequalities affecting both participants and research partners. This article describes the ethical challenges of international gender-based violence research from the perspective of Kenyan researchers and organizations and identifies recommendations about how to prevent them.

They asked: “can we have some survivors? […] and then they pushed to the extent that they asked: ‘can you take us to the place you were raped? So you can share your stories from there and we can film.’”

Although this example given by a representative of a recovery center for gender-based violence (GBV) survivors might refer to media reporting rather than research, the anecdote poignantly reflects the potential risks and harms to survivors involved in research and reporting on GBV. Hearing GBV survivors’ stories is important since it allows for understanding the lived experiences of violence, the effects it has on survivors and societies, the response or lack of response to those effects, and how GBV can be eliminated. This is an important complement to the common preference, especially among policymakers, for quantitative evidence of (sexual)violence against women, which tends to isolate this violence from the social, cultural, and political context in which it takes place, and, therefore, at best leads to a partial understanding of its causes and experiences (Boesten & Henry, 2018). Nevertheless, the quote indicates that there are several risks involved in research with survivors of GBV, especially if research is done by researchers from high-income countries (HICs) who are not always familiar with local contexts and cultural sensitivities, and whose geographical and socioeconomic backgrounds produce power inequalities between these researchers and participants from low- and middle-income countries (LMICs). If these risks are not heeded, research might end up producing more harm than good for participants.

Based on interviews with individuals who have been involved in diverse ways in research with or support for GBV survivors in Kenya, this article describes some of the most pressing ethical concerns in regard to undertaking cross-national GBV research. These risks are present not only for GBV survivors participating in research, and for organizations accompanying them, but also for researchers from LMICs who are often hired by researchers or institutions from HICs to conduct parts of this research. Their experiences illustrate different inequalities that may make research an exploitative experience for Kenyan participants and researchers. The findings of our research deal both with our participants’ views of Kenyan GBV survivors’ experiences as research participants, and with their own experiences as partners in research partnerships. We thus refer to two levels of participation in our empirical data, as illustrated in Table 1.

**Table 1.** Overview of Themes and Codes.

Analytical theme	Descriptive theme	Number of participants that made reference	Level of participation: research participants or partners
Conceptualizing and developing the research project	Risk of conducting research that only benefits the researcher	4	Both
	The importance of transparency, accountability, and trust	2	Both
	Need for capacity and resources to do research	6	Partners
Collaboration throughout the course of the project	Encourage skills development and expertise exchange	3	Partners
	Research as a collaborative process	4	Partners
	Being responsive to needs and diversity	2	Participants
Ethics of researching gender-based violence (GBV)	Involving and engaging survivors and the community	6	Participants
	Compensating participants and local researchers	2	Both
Emotional impacts of research participation	Research duplication and participant exhaustion	2	Participants
	Conducting research sensitively	2	Participants
	Harm and distress experienced by those participating in research	4	Both
Dissemination, publication, and impact	Getting feedback on findings from participants	3	Participants
	Sharing accessible information	2	Both
	Research as input to help develop programs	4	Both
	Research results to inform media and policy	7	Both

The article will proceed by briefly outlining the main phenomena described in the literature on ethics in cross-national GBV research, in particular the risks of re-traumatization, exploitation, and research fatigue. It then moves on to describe the methods and context of the research. Its findings are grouped among different phases and aspects of the research process: We first discuss wider issues concerned with the conceptualization and development of research projects, which more often than not is an undemocratic exercise. We then discuss the main challenges in conducting research in a way that responds to participants’ needs and perceptions, describing the ethical and emotional impacts of research and how these, as well as the risks of research, are frequently unfairly balanced. Finally, we describe the ways in which research is and can be disseminated, another area which frequently highlights international inequalities. In a qualitative research tradition, we place our findings in the context of and in dialogue with similar research. The discussion then summarizes the power inequalities that are at the heart of many of the unsatisfactory experiences of Kenyan participants in international research collaborations, to conclude by making recommendations to overcome the main challenges of cross-national GBV research.

## Ethical Challenges of Researching GBV

Sexual and other forms of GBV often have serious consequences for the survivors. Apart from physical harm, GBV has emotional repercussions such as fear, trauma, and shame, as well as the psychological impacts of threats and intimidation faced by survivors and their families, while sexual violence particularly can produce severe social consequences. Survivors of sexual violence are often considered impure or promiscuous women, frequently leading to stigmatization or even rejection by their families and communities, which in turn condemns survivors to poverty (Boesten & Henry, 2018; Duggan & Abusharaf, 2006). As a result, survivors are often reluctant to talk about their experiences, as a way to protect themselves from the social backlash they face when their stories become public, as well as from potential repercussions by perpetrators. Silence can therefore be a protection strategy (Drumond, 2016; Eastmond & Mannergren Selimovic, 2012). This explains why survivors can be hesitant to participate in research. First of all, participating in research can cause re-traumatization by stirring up painful memories (Sharp, 2014). It can also put survivors at risk, if the perpetrators of the violence they suffered are still close to them, or if their communities find out about what happened to them. Survivors might struggle to see the benefits that research participation will bring, as academic knowledge production rarely has tangible results for them. Processes to change policies are lengthy, while the risks to survivors’ safety and well-being are acute (Boesten & Henry, 2018).

As a result of this tension between the potential benefits and risks of research with GBV survivors, an additional concern is research becoming an exploitative or extractive experience for participants. Characterizations of “drive-by” or “helicopter” research reflect the ways that survivors of violence, Indigenous peoples, and community organizations have experienced involvement in the research process, with outside researchers entering a community to collect data to meet their own needs with little concern for participants’ cultural context, practical and emotional needs, or the long-term impact of their involvement (Horowitz et al., 2009; Kral, 2018; Williams, 2004). Inviting women and girls—and men and boys, who can also face GBV—to break the silence about having experienced GBV can make them feel used by researchers or journalists, who advance their careers by portraying the suffering of “vulnerable” groups of women without any tangible results for these same women, who often face conditions of marginalization and poverty (Boesten & Henry, 2018; Olujic, 1995). Participating in research—or journalistic reports—can thus lead to a feeling of research fatigue. This refers to a reluctance to participate in research because of previous research experiences, often after long-term or repeated participation in research projects. Research fatigue can become particularly pronounced when there are no perceived changes as a result of the study, when change cannot easily be linked back to research participation, or when promises about research impact have not been met (Boesten & Henry, 2018; Clark, 2008; Mwambari, 2019). This can produce feelings of disempowerment and instrumentalization.

These exploitative and harmful research practices, and the resulting research fatigue, are frequently stronger in geographical areas and on topics that are “over-researched,” which have become the subject of repeated or continuous attention of researchers (Boesten & Henry, 2018). Over-researching is likely to occur in settings or on topics that become a “hype,” which refers to a phenomenon that, temporarily or more permanently, attracts an extreme level of public attention, either by the media, political actors, or aid agencies (Hilhorst & Douma, 2018). Academic research is also influenced by such hypes, as they spark funders’ and researchers’ interest (Boesten & Henry, 2018). Sexual violence in conflict can be considered such a hype. On a smaller scale, as we will discuss in this article, the sexual violence in the post-election turmoil in Kenya can also be understood as a hype, attracting a flurry of academic research and humanitarian and journalistic reporting. Such hypes lead to over-researching and repeated research participation by a sometimes limited number of participants whose stories are considered most “attractive,” and who, therefore, are expected to recount their experiences numerous times (Mwambari, 2019). This exposes participants to the risks of re-traumatization and feelings of disempowerment and exploitation.

Although concerns of re-traumatization have been addressed in international guidelines and research (see e.g., WHO, 2016; Ellsberg et al., 2001), there is very little attention to the specific challenges produced by additional power inequalities when this research is undertaken in LMICs by research institutions from HICs. In this research, feelings of being instrumentalized or even exploited by research are not limited to research participants but are also frequently experienced by the researchers from LMICs who are hired to collect data in the field. They are often called “local researchers,” which is in itself quite telling, because it denotes a difference in position and power between “local” and “international” researchers, with the implication that “local” is in some way inferior. This different valuation is also frequently reflected in the remuneration of researchers from LMICs, who tend to receive lower salaries while being exposed to much higher safety risks (Cronin-Furman & Lake, 2018; Mwambari, 2019). As a result, they may find themselves in an uncomfortable position, since despite not always being able to influence the research practice, they are the ones most directly facing the participants. Sometimes researchers from LMICs even encounter the same participants in subsequent research projects and are confronted with the lack of results from earlier studies (Chiza Kashurha, 2019). In light of these issues, this article addresses the specific and compounded ethical challenges of the intersection of cross-national research and GBV research.

## Methods

The aim of this research project was to explore the nature of ethical research practice in research concerned with GBV in Kenya, as illustrative of other LMICs, particularly exploring experiences in participatory research and in international research collaborations. This was done in light of a broader research project which intended to establish a global standard for research engagement with survivors of GBV in LMICs, to contribute to better research with, and outcomes for, survivors. We provide more details on this project in the final section of this article. Ethical approval to undertake the study was granted by the University of Birmingham Ethics Review Committee (ERN_18-19280). All interviews were conducted in June 2019. We recruited a diverse sample of representatives from organizations in Nairobi, Kenya: an academic researcher; an independent researcher; a health research organization; two representatives of a consulting firm working on social, health, and gender issues; an activist; a grassroots shelter for women and girls who are survivors of sexual violence; a recovery center for GBV survivors; three representatives from two NGOs working on women's rights and GBV from a health perspective; and two NGOs working with women from a more legal perspective. Access to the sample was mainly through a purposive recruitment process, with potential participants contacted via an organization called the Wangu Kanja Foundation, with whom we had a prior relationship. We then implemented elements of snowballing, whereby further participants were contacted through recommendations of other interviewees. Nine interviews were conducted face-to-face in a field trip visit to Nairobi and three interviews took place via Skype after the visit. All interviews were conducted in English by two members of the research team. Participants were asked about their work, their experience with doing (participatory) research on GBV and any ethical issues they experienced, their experience with and desire to collaborate with international partners, their ideas about the form that cross-national research should take, which types of research outputs would best serve their work to generate change for survivors of GBV and their priorities for future research projects. All audio recordings of the semistructured interviews were transcribed verbatim. These transcripts were analyzed using thematic analysis (Braun & Clarke, 2006) and inductively coded line by line using NVivo 12. An initial analysis was conducted, where first, descriptive themes were coded, and, following subsequent readings and analysis of the transcripts, analytical and higher-order themes were created (Table 1). These themes were then discussed and further developed. This article discusses the themes that focus on ethical research practice on GBV in the Kenyan context.

There are some limitations to this study. First of all, the participant sample is not large enough to enable generalizations. Our insights however do speak to existing literature on participation and fatigue in GBV research, adding the perspective of cross-national research collaborations. Second, our interviewees were all based in Nairobi and therefore their views are not necessarily reflective of organizations in other regions of Kenya. Finally, the research was conducted by white researchers from HICs, and reflects their interpretation of the views and experiences of the participants. Although the analysis was shared with participants, who were invited to provide feedback, the analysis may have been influenced by the researchers’ positionality.

## Experiences With GBV Research in Kenya

Most of the participants we interviewed did not implement their own research projects--not even in the case of the academic researcher—reflecting global inequalities in terms of research funding, as African academics often have little access to funding to conduct academic research (Dodsworth, 2019). Nevertheless, many of the interviewed persons and organizations had been involved in research indirectly, for example, having been approached by HIC researchers looking for GBV survivors to participate in their research. In this way, organizations are often asked to act as gatekeepers for international research. Others had direct experiences in undertaking research themselves, frequently as part of international research projects with HIC principal investigators (PIs), or on projects funded by international NGOs. The following discussion therefore draws on experiences both as gatekeepers and as researchers. The data are presented in relation to several key aspects of the research process, starting with its conception and development, then discussing the ethics of data collection, and finishing with its dissemination and impact. Participants’ experiences illustrate both poor and best practices. While these examples are based on the Kenyan context, they offer a point of reflection for research generally conducted in LMICs on sensitive topics.

### Conceptualizing and Developing the Research Project

The tensions between the needs and priorities of researchers from HICs and those of survivors, organizations, and researchers based in LMICs are evident from the conceptualization stage of the research project. Four interviewees identified the concern that rather than starting off by exploring the research priorities of participants, research projects are often initiated by international PIs or donor agencies, who thus define the goals of the project and set the research questions. As a result, research questions may not reflect the interests and priorities of the African partners or the research participants. Six interviewees pointed out the crucial fact that organizations and researchers are frequently reliant on HIC partners to bring in funding. This however means they have to adapt to international research and policy agendas. This dynamic can create a power imbalance within the research partnership, since researchers from HICs, consciously or unconsciously, can exploit organizations or academics that may be desperate for work or reliant on external funding. An academic researcher explained:

Of course we do research but to be honest there is no research of sexual and gender-based violence in the university because there is no funding and you really have to rely on funding from UK universities. […] if you never get funding from international organizations, it gets really hard so I think funding is one of the predicaments of the university: if you don't get the funding you can't carry on. (Interview 4 June 2019)

The impact of the funding regime on the type of research that is conducted was apparent in several interviews through examples of topics of international interest that had dominated the research agenda. The academic researcher mentioned that there was a flurry of research on a specific form of violence: “The type of SGBV that the organizations have worked on is large-scale violence, generally during post-election periods” (Interview 4 June 2019). An independent researcher echoed this: “One of the people asked me, ‘Why didn't you sit with us and develop this research with us?’ and even asked us, ‘Is sexual violence the issue?’” (Interview 13 June 2019). She also mentioned the intense research activities in the slums as another example of an area that is over-researched: “Right now in the slums, we just say the word research, and no one listens to you. […] Especially in Nguru, people are tired, because they get asked questions, but they don't get any feedback” (Ibid. Interview 13 June 2019).

These examples reflect how research projects in Kenya are often responding to hypes (Hilhorst & Douma, 2018), which can result in duplication of projects and do not necessarily reflect participants’ priorities. The focus on international priorities driven by funding inequalities is not unique to Africa; it is also seen in research in the Middle East, where research assistants feel exploited by the many international research projects on migration, which are presented as “noble” (Sukarieh & Tannock, 2019). This approach maintains relations of global inequality in which LMIC participants and researchers must adapt to HICs priorities, reminiscent of colonial times in which Africa was used as a living laboratory (Dodsworth, 2019).

To break this cycle, participants recommended much earlier involvement of community organizations and researchers from LMICs, ideally at the funding application stage, to ensure that the areas of research focus can respond to the needs of those most affected. Communities should be asked what their concerns are, what they feel researchers should study, and what changes they want to see as a result of the research. A representative from a women's NGO explained how her organization seeks to involve the local community from the outset to ensure greater legitimacy for the project:

As we are designing what the study would look like and the parameters it would cover, we always like to engage the community, because at the end of the day they are the ones who will consume the information. […] The resistance towards the results that you might get would be much higher if you have not involved them from the word go, in terms of designing the tools that are being used, deciding on the sample size, and identifying the geographical area of the study, and then also coming to an understanding of what is going to be done with this information. (Interview 3 June 2019)

Participants emphasized that during this process one should respect local timescales and procedures. This means that communities and participants should be given time to digest the information before deciding on participation, rather than just running a workshop and expecting people to respond there and then. For a member of a women's rights organization, this requires a more continuous process of communication with the participants: “if you are starting a project today, you can go for the first time and explain what the project is, and give them time as a community to discuss it amongst themselves, and then go back and hear what they have to say” (Interview 6 June 2019).

At the same time, it is important to manage expectations, so that survivors and community members have a realistic sense of the remits and limits of research. Although researchers might aspire to bring about positive change, this may depend on political decisions that are not within their control. If expectations are not managed, the research process may create disillusionment. A women's rights organization representative explains: “There are a lot of misconceptions and you need to be clear. [Otherwise] they may feel like they were lied to or that it is pointless. You need to clearly explain what research can and cannot do. Explain that you are not the government, you can't change everything” (Interview 6 June 2019). This illustrates the point highlighted by most participants about the importance of transparency in the research process. Transparency means that researchers share their purpose and approach, which can facilitate a conversation about how and why the research is being conducted, strengthening ethical integrity and practice. Transparency also signals to the participants that they are taken seriously by the researchers, that they are not used simply as sources of data but have a say in the research and can hold the researchers accountable. A member of a women's organization explains: “They want to know exactly what are you doing with this information, so it is important that that is clear from the word go and you can account later: you said you were going to do this with this information and now we see you doing this—where has the shift happened and why?” (Interview 3 June 2019).

Furthermore, particularly with interviews, understandings and interpretations can differ, so at the very minimum it is important to reach out and ensure that the analysis is in line with what was intended by the participants. Without this ongoing engagement, participants may feel exploited by the process, according to a member of a women's rights organization:

[You need a] system with which you are able to explain what this research is about, how I intend to use this information, and if it is a year long's work you should be able to come back after that time and say, "We have achieved what we wanted and this is how your input as a community was able to assist the research." […] [Now] it's a cycle of people feeling used and not feeling that they’re getting what they need. If things were done the right way, it would be beneficial to everyone. (Interview 6 June 2019)

Engaging with the local community and survivors, therefore, should not be seen as optional or a one-time event, but as a continuous process of ethical, transparent, and accountable research collaboration. This will help build trust and create more equal relationships.

### Collaboration Throughout the Course of the Project

The early stages of the research process are crucial to establish an equal partnership and, according to a representative of a health research organization, agree that “the extent of the role that you are expected to play from the start” (Interview 4 June 2019). Understanding these expectations also enables partners to make an informed decision about participating in the project. For representatives of a women's organization, the notion of equality between all partners in a collaboration is fundamental: “It feels like a true partnership if it is something we can agree on which is of value to both of us rather than having someone come and just be around, do a bit of data collection” (Interview 3 June 2019). Even where organizations or individuals may lack capacity in particular areas, they still want to be involved in the entire research process, not just as data collectors or gatekeepers. A representative of a health rights organization explains: “[If] we were conducting the research in partnership we would appreciate to be involved in all the different stages if possible, also depending on the capacity we have ourselves. […] For example, we don't have capacity to do quantitative data analysis, but it would be nice to still be involved” (Interview 21 June 2019).

Participants in our research described that if a research partnership is initiated and maintained in a transparent and equal way, it can be a very valuable experience for *all* partners, and provide better quality data and a greater likelihood of impact. Often, it is considered that the advantages of such partnerships are greaterfor the African partners, who benefit from the funding and the methodological expertise of researchers from HICs. A member of a health research organization described an enriching collaboration early on in her career: “We got to engage with academics from all over the world so we got to share lessons, develop proposals on similar themes, so it was a very rich learning experience for me as a young scholar” (Interview 4 June 2019). An academic researcher described the importance of learning about academic ethics procedures, which many NGO researchers are less aware of:

I don't think we are doing all these interviews by the book […] and actually look at the ethics. Of course, when doing the research you would sign the consent forms but I don't think they adhere to the proper procedures and perhaps such partnership can help people in organizations to actually know what is supposed to be. […] [NGOs have not had] the proper training about how to conduct research with people who are victims or survivors of sexual violence, so the partnership could be even just giving people information on how to handle victims in a proper way. (Interview 4 June 2019)

International research partnerships, however, do not only benefit researchers from LMICs; HIC researchers too have much to gain from working together with Kenyan researchers. According to members of a consulting firm working on health and gender issues, collaboration can be a real “two-way exchange” of expertise (Interview 7 June 2019), since African researchers and practitioners have rich knowledge about the cultural and political context. The director of an organization supporting GBV survivors explains: “We complement each other. I have the country background, the grassroots expertise, you might have the academic background; you gain, I gain. At the end of the day, we present a paper which is all inclusive and have factored in the different options you are supposed to look at” (Interview 4 June 2019). A women's organization pointed out that, especially in research on sensitive issues such as GBV, this contextual understanding is vital: “You need to understand the different dynamics in different communities. There are communities where you cannot have a conversation with men and women together, or there are communities where you have to take certain religious customs into consideration and ethnic dynamics that you have to be aware of” (Interview 3 June 2019). This contextual knowledge can help avoid bad research practices, such as asking culturally inappropriate questions that can be offensive to the participants (Mwambari, 2019). Moreover, the director of an organization supporting GBV survivors explains that local organizations often have contacts that are crucial for the success of a research project: “We are at an advantage because we have been working on this for a very long time. We have the networks, contacts, individuals who we can link the research institutions to or we can recommend having conversations with” (Interview 4 June 2019). Unfortunately, our findings echo tendencies apparent in cross-cultural research on non-gendered violence, that such local knowledge is not always valued equally with academic knowledge, even though it can be crucial to decide which methods are most adequate or to analyze the data in all its depth (Cronin-Furman & Lake, 2018; Mwambari, 2019).

When a research partnership respects and values those different skills and assets, the experience can be “fantastic,” in the words of a representative of a health rights organization. She described a partnership in which there was “a lot of dialogue and we can push back respectfully and say, ‘that is not relevant’ and ‘that won't work in this context’” (Interview 21 June 2019). A good research partnership should consider the strengths of the various individuals involved and value them equally. Although researchers from HICs might have more access to funding, partners from LMICs are more likely to have language expertise, knowledge about how to best recruit participants, and contextual knowledge that is crucial for data analysis. This enables an exchange of skills and knowledge that can be highly beneficial. The partnership should also identify which skills people would like to develop and how they can learn from each other, in a two-way process instead of the common assumption that capacity-building only runs North-South (Dodsworth, 2019). Ultimately, all people involved in the research can then learn new skills throughout the process and become better researchers as a result.

### Ethics of Researching GBV

Ethical research conduct on a sensitive topic such as GBV in LMICs includes reflecting on the process of data collection and analysis. Many participants believed that there were serious issues with the approach and methods of research. They confirmed prior research practices where researchers were interested in listening to survivors directly, prioritizing those who have the most "attractive" stories, or those who are best able to articulate their stories in eloquent and engaging ways (Krystalli, 2019; Mwambari, 2019). This, however, means that the same group of people are engaged, for whom it can be very tedious to be interviewed repeatedly, often being asked the same questions. At the same time, another group of people is excluded, their experiences and needs not being heard. Since a similar narrative is being explored by speaking to the same people, there is a risk that other issues which may be crucial to large groups of people are overlooked (Boesten & Henry, 2018). This means that policy recommendations resulting from a research project may only be based on a certain version of the events, and do not respond adequately to the diversity of experiences and needs.

In the interest of generating media attention for their research, in most cases sparked by a genuine interest to produce policy impact and change in the world, researchers do not always show sufficient sensitivity or understanding of who their participants are, and what the impact of repeated research participation is for them. This pattern of over-researching certain groups of participants is reinforced by the lack of willingness to share research results. As an independent researcher indicated: “If you don't share your report then it's not available, so people repeat the research” (Interview 13 June 2019). An academic researcher suggested that researchers and local organizations better “co-ordinate their work and ensure that once one organization has interviewed, they don't go back to the same survivor and ask them the same set of questions” (Interview 4 June 2019). This echoes calls for more reflection on whether first-hand accounts of direct survivors’ experiences are always necessary (Boesten & Henry, 2018).

Other researchers have pointed out that some local communities and survivors’ organizations have adopted their own ethics protocols or memoranda of understanding, to which researchers should adhere if they want to undertake research with them (Dodsworth, 2019; Madlingozi, 2010). Some of our participants admitted having refused research collaborations. A representative of a women's rights organization suggested that sharing such experiences can help individuals and organizations to say no to poorly designed or harmful demands:

I think maybe sometimes we don't share how we want things to be done. So, the perpetration [of unethical research] continues because we keep quiet about it, we don't say “Look, this is wrong.” Just because you have money and funding it is not right to do it this way or expect the victims to just be there waiting for you to turn up and say, “I need this information.” I had to learn; other people can learn. (Interview 6 June 2019)

Making sure that research participants obtain and claim more agency in deciding whether and how to participate in research is a crucial step to making research more ethical and research collaborations more equal.

Almost all participants stressed the need to give a voice to survivors and engage with them. Although most participants did not have experience undertaking participatory research, they saw the benefit of using this approach to facilitate the active participation of survivors. A representative of a health research organization explained their current approach:

We are very big on ensuring that the voices of survivors are extremely important; there's no point in doing this if we’re not going to listen to them. They often have really great ideas about what we should and shouldn't be doing. So their voices and needs are definitely incorporated. Although I can't say we are doing community-based participatory research, I can see the importance of it. (Interview 4 June 2019)

However, the timescales and aims of some international projects can be a barrier to meaningful participation, as relationships need to be built up over time. Pressures on researchers from HICs are partly to blame for this, as they are expected to obtain research funding for large projects and publish in top journals to respond to academic evaluation processes. This leaves many HIC academic researchers with little time for doing the actual research on the ground (Sukarieh & Tannock, 2019). As a result, local organizations are put under pressure to produce results, as a woman's rights organization representative poignantly illustrated:

We had a group from outside the country who reached out to say, “We are working on this document, we would like to get the input of sexual violence victims in Kenya but we only have three weeks.” It seemed like it was a big project which had been going on for a year. […] The way it was presented was like there was no time, you have to do this. I chose not to participate in that meeting, not because I thought it was not useful, but because I felt it was not being done in the right way. (Interview 6 June 2019)

Something that is discussed more widely in relation to research ethics is whether or not research participants should be compensated. It is generally not seen as ethical practice to compensate participants, as this might undermine their objectivity and impartiality. Nevertheless, when participants find themselves in difficult socio-economic situations, it can be hard for researchers not to offer anything for investing time and providing sometimes essential information (Cronin-Furman & Lake, 2018; Mwambari, 2019). Although our participants stressed the importance of compensating individuals for their time and (travel) expenses, they also pointed at the potential perverse effects of this. Particularly in highly impoverished areas, this can make survivors become dependent on research, or lead to individuals falsely claiming to be GBV survivors as they are in need of money. An academic researcher explained:

Some people engage in meetings because they know at the end of the day they get some money for transport investments, meal allowance. So you can find the true victims and the fake victims because of money issues. I remember last year […] almost 100 more women came in for the conference when we had only called for 50 or so, but when news spread that they could be reimbursed for transport, 100 more women stood by the gate wanting to come in. It is a fine line between rewarding people for the time and money they incur but not creating this dependence […] that will affect the type of data, because you could get skewed data. (Interview 4 June 2019)

This is a real dilemma that can create ethical tensions, especially for the African research partners who are faced most directly with survivors’ difficult situations. The director of an organization supporting GBV survivors also points at the need to recognize the role of organizations that do not participate in the research as such, but have a gatekeeping function: “We have all these international organizations coming into the country; how do they compensate time for people who are providing or creating venues for them to access that whole chain? […] You are assuming they are on a salary, but what if they are not, how do you compensate their time and opportunities?” (Interview 4 June 2019). These different aspects of the everyday practice of research, both in enabling the participants to be contacted in the first place and then of enabling participants to take part in it, often imply costs. These costs are taken for granted by HIC researchers, but they can be crucial for partners and participants in LMICs. This is just one more reflection of the inequalities present in international research projects. Researchers must consider them and prepare for them, as should funders, who are often not interested in funding the everyday costs of research or its overhead and administration costs (Dodsworth, 2019).

### Emotional Impacts of Research Participation

A key concern raised in relation to the conduct of research was the emotional impact of participation for both survivors and researchers. As explained above, research driven by international priorities tends to focus on a limited set of topics, and engage a certain group of survivors, often repeatedly. An academic researcher described the functioning behind this:

This person [gatekeeper] might always give you the same ten people, because this person is looking for reliability. Maybe you know that this [victim] is reliable and because you know she will come, you will find the same faces and the same people turning up at meetings. […] We only focus on those people who are able to articulate. (Interview 4 June 2019)

This leads to certain groups of participants being invited over and over again to participate, and thus easily becoming over-researched and drained. This, in turn, can lead to research fatigue, particularly when the participants see little benefit from the research: “Someone will tell you: ‘You are the 50th person interviewing me; what are you going to do differently?’ People don't see any results, so they’re just tired from the process. It's like they’re being used and they are not getting anything out of it” (Ibid. Interview 4 June 2019). According to a women's rights organization employee, survivors’ stories are sometimes just an afterthought, meant to illustrate the points made by researchers in projects that do not actually center the participants’ needs and experiences: “I felt the way it was done, I really thought like the victims were an afterthought. Maybe someone said, ‘you can't have a report without their input’, and they thought, let's go and find some” (Interview 6 June 2019). This shows that the dynamics of research by HIC researchers in LMICs, with its heightened global inequalities and insufficient attention for local timeframes and needs, increases the risks of exploitation and research fatigue among survivors.

GBV research leads to risks not only for participants, but also for LMIC researchers, for whom researching GBV can be extremely distressing and also dangerous. It is well-established that hearing repeated accounts of violence can lead to secondary traumatization (Campbell, 2002). A member of a women's rights organization acknowledged this:

When I worked on the post-election violence case in 2007, it was so traumatic. Every day you would sit and speak to victims. Then we went back to the office and we just couldn't work. After some time, a counselling support system was implemented and then we realized we were not fine. So, that helped, but even after I left [the organization], in civil society the most we ever do is sit down and speak amongst ourselves and say, “Ah, we’re tired.” But I think we need more support. (Interview 6 June 2019)

This shows that LMIC researchers do not only collect the data, often requiring long working hours and sometimes facing safety risks, but also take on the burden of the emotional labor of research, while the emotional aspects and impacts of researching sensitive topics are literally far away for the HIC researchers (Sukarieh & Tannock, 2019), thus adding another layer of inequality in international research projects. LMIC researchers are generally not recognized for this additional burden, and instead tend to receive much lower salaries. They often feel they have no other option than to dance to the tunes of those providing the funding (Mwambari, 2019; Sukarieh & Tannock, 2019). This can lead to feelings of exploitation and alienation for these researchers, who feel instrumentalized and exploited, simply treated as data collectors under extreme time pressure (Sukarieh & Tannock, 2019). Counseling and other mental health support can be helpful. A grassroots women's rights activist pointed out, however, that “it's extremely expensive in this country and we have less than 300 mental health experts serving 50 million Kenyans” (Interview 4 June 2019). This means that international research projects should budget for this support, as it cannot be assumed that researchers from LMICs can access it themselves.

### Dissemination, Publication, and Impact

As the research process comes to an end, there can be tensions between different perceptions of meaningful dissemination and impact. The end of the data collection phase should not mean the end of the research collaboration. On the contrary, the dissemination of the research findings and the generation of impact based on them are a crucial part of the research, as is illustrated by the frequency with which this topic was mentioned in our interviews. Dissemination should start with the essential step of presenting the research results to participants and stakeholders in a language that is easy to understand for them, and inviting them to provide feedback. The director of an organization that supports GBV survivors explained: “Once we have done a research document, we have been going back to the people who have contributed and asking: Is this a true reflection of what you said?” (Interview 4 June 2019). A women's organization echoed how this strengthens the validity of the study:

One of the most important parts would be the validation of the report; the stakeholders and the people who are involved in the collection of the data in any way come to the table and do the presentation on the findings, and at that point they can feed back: “Ee don't think this represents correctly what we’ve said.” And we also have the participants who were in the study in the validation and they can say, "No, this is not what I said" and give feedback, and that's very important. (Interview 3 June 2019)

However, this process of validation and results sharing does not always take place, as evidenced by the experience of an independent researcher in an international research project: “It was such an uncomfortable place; we could not even speak back. I asked the organization, ‘Can we go back and really sit with them and show them what we found?’ but they were like, "No, this is our report, let's roll the program’” (Interview 13 June 2019).

This shows that in many cases, researchers from LMICs are in an uncomfortable position, being stuck in the middle between HIC researchers or institutions who bring the funding and therefore decide on the research, and the participants who demand to see the results. Some participants in our study held the perception that HIC researchers were exploiting communities for financial or professional gain, and the failure to share results reinforced the notion that those who benefitted most were the HIC researchers. This reflects a lack of transparency surrounding the purpose and outcomes of the research, according to a representative of a GBV recovery center: “As soon as they have their results then they go. We need to make sure we are benefitting the organization or the community, you know” (Interview 5 June 2019). According to a representative of a women's rights organization:

The locals feel: people get information from us and we never see the results ever again. No one comes back to us and says. “Remember the meeting we had last year? This is the result, and this has been shared in this and this place.” Some feel that people are benefitting financially. Most times it's not true, but I don't blame them for feeling that. (Interview 6 June 2019)

For academic researchers from HICs, successful impact may be measured by publications and citations for an academic or broader international audience, but local communities are likely to seek more concrete outcomes. An academic researcher shared the example of the many studies on the post-election sexual violence, which did not result in tangible change:

[I]t's been almost 12 years since the violence broke out and we have seen nothing happen. So we find that most of the victims have not been compensated, some have died, some of them are still sick; because of the violence they experienced their lives have deteriorated, their economic situation has changed, their family left them. We are still waiting for a success story. (Interview 4 June 2019)

Even where a research project is aiming to create meaningful change, international research can still reproduce colonial dynamics, with international partners drawing out policy and practice recommendations without involvement from survivors and the local community. In these cases, there is a risk that the solutions proposed, even if well-intentioned, have limited legitimacy and fail to respond to the key priorities of those most affected. According to a women's rights organization, this leads to “a situation where policies are implemented as a result of the research, with the community saying,this is not what we want, who asked for this?” (Interview 6 June 2019). The participants, therefore, should be involved in discussions about what they want to come out of the research, rather than the researchers speaking and deciding for them. A representative from a women's rights organization reports that the contrary is often true:

In our engagement with victims, the one thing that over the years they complain about is that they feel like an afterthought. When it comes to discussions about policies or any form of assistance or reparations that victims have a right to access, they are usually the last to be told, so at the beginning of the process people [researchers] will start with government officials or NGOs and then people sit down and come up with all these things that they think victims need. (Interview 6 June 2019)

Beyond the step of validating the research, participants also spoke of the importance of making research findings accessible to all stakeholders in the community, and not just to academic or international audiences. To make an impact, research should produce more diverse outputs than just academic articles, which have limited long-term and tangible impacts for research participants, as a representative of a GBV coalition explains:

I think it is important that we anchor research on an academic platform, […] but in the way it is packaged and delivered to different audiences, we might make proposals to say maybe we can do a video or a documentary, or a press brief, or we can have dialogue around this in a way that can reach the communities and different audiences that may not necessarily digest an academic piece of research. (Interview 21 June 2019)

In looking for more diverse ways to engage different audiences, there is a crucial role for researchers and civil society organizations from LMICs, like this health rights organization: “It's about offering practical recommendations because that's often a challenge where the researchers are so academic, you need someone else to be able to assist, to show you how this can be useful for the survivors and the people working with the survivors” (Interview 21 June 2019). Disseminating research findings more widely requires producing locally accessible and meaningful research outputs, avoiding jargon, and translating the findings into different local languages so that survivors and their organizations and communities can use the findings for their own activities, such as advocacy. The director of an organization which supports GBV survivors explains that this is not only part of ethical research practice, but also increases the potential for impact:

We are helping the survivors to understand the violations and the findings and then we help them have that conversation with the county or national governments, media houses, civil society organizations. So it's about building their capacity so they are able to negotiate and have conversations with people who do not understand the issue. (Interview 4 June 2019)

Participants also discussed the importance of engaging with local media. While international media are often a higher priority for HIC researchers, engaging local media is a potential route to share findings and promote positive change to local communities. Local media can have considerable power to engage and influence the public discourse, which can help to challenge public attitudes towards GBV. This can also contribute to advocacy for change with local policymakers. Collectively planning and implementing a successful impact strategy requires time and resources, which needs to be considered at the research planning stage.

Within the process of discussing and producing diverse research outputs, including academic articles, media, and policy pieces, it is important that the contributions of all researchers in the process are acknowledged equally, rather than only those of the researchers who did the actual writing. Unfortunately, as other research has pointed out, more often than not researchers from LMICs are excluded from publications, even though they sometimes did not only do the fieldwork but also much of the writing (Cronin-Furman & Lake, 2018; Sukarieh & Tannock, 2019). Including African researchers in publications is not only an elemental aspect of managing equal and ethical research partnerships, but also a way of contributing to the decolonization of academia, since African authors tend to publish most in African journals and less in internationally leading journals. This tendency is even stronger on issues around gender, where female scholars from LMICs are severely under-represented in leading, HIC-based journals (Dodsworth, 2019; Medie & Kang, 2018). Our research in Kenya showed that co-authored academic publications are of interest to many researchers there, as a way of showing the credibility and legitimacy of their work, to learn from academic researchers, and vice versa. A representative of a health research organization pointed out that “the people on the project are able to give valuable contributions to peer review publications or the final report” (Interview 4 June 2019).

## Overcoming Power Inequalities in International GBV Research

The dominant theme which ran through the experiences of our participants was the disparity of power between survivors, organizations, and researchers based in Kenya, and researchers from HICs. Research relationships were often extractive, with outside researchers imposing their timescales, methods, and priorities rather than building long-term, collaborative relationships for collective benefit. The power dynamics were particularly reflected in economic and epistemic inequalities, and the imbalance of risk and reward. Economic inequalities frequently resulted in a focus on topics that were of interest to those financing the project and to a wider international audience, rather than to those most affected by it. The international demand for research into short-term, high-profile hypes often leaves little room for the kind of nuanced, long-term projects that are needed to bring about meaningful change for survivors. This also leads to over-researching specific issues, resulting in research fatigue among survivors and organizations, while other topics remain neglected. Economic inequalities are also apparent in the different rates of payment for LMIC-based members of the research team compared with their colleagues from HICs. From an epistemic perspective, the findings demonstrated the lower value that tends to be attached to knowledge held by research partners in LMICs. Their specific knowledge of the local context, survivors’ needs, and local priorities for research and service provision are frequently given a lesser status than the theoretical and ethical frameworks imposed by outside researchers and institutions. This epistemic imbalance is also reflected in the make-up of the research team, with partners from LMICs often positioned in subsidiary roles, and capacity-building generally involving researchers from HICs providing training and experience to local partners rather than LMIC partners sharing their expertise or a mutual exchange between all those involved. The third area of inequality that ran through the findings was the imbalance of risk and reward. Participants described the outsourcing of emotional labor to researchers from LMICs, making them responsible for managing the emotional and practical risks to survivors along with their own risk of re-traumatization, while the benefits were weighted towards the researchers from HICs. Purposes and outputs of research may also be viewed differently among HIC and LMIC researchers. Collectively addressing these power inequalities is important from the perspective of ethics and justice, but is also a vital way of ensuring the quality, relevance, and impact of research outputs. Ultimately, ethical research practice should not be seen as a burden but as a responsibility to the community. Moreover, engaging in ethical research is far more likely to produce reliable and valid data that have a meaningful impact for the survivors and their communities.

Although guidelines exist in relation to GBV research, participatory research, and international partnership, there are so far no guidelines which highlight the centrality of participation to address the specific ethical challenges of international GBV research across HIC-LMIC partnerships (Thomas et. al., 2020 ). This is a clear shortcoming. This article has identified how the global inequalities inherent in international research add specific challenges to the already existing risks of GBV research, not only for participants but also for partners from LMICs. These risks are not sufficiently addressed by institutional ethics boards either, as they tend to focus too much on “procedural ethics,” aimed at managing safety, confidentiality, and consent, and less on ethical research practice throughout a project (Guillemin & Gillam, 2004). To address this gap, we have developed the ENGAGE (Ensure No “Grab And Go” Extractive Research) guidelines (Bradbury-Jones et al., 2020) , in collaboration with partners in Kenya, Uganda, and Guatemala based on the present research, together with a broader review of the evidence on best practices and an international discussion workshop.

The ENGAGE guidelines are centered around four key principles: research should respond to the sensitivity of the topic; be aware of, and mitigate risks to, researchers and participants; be designed and carried out in a collaborative way throughout the entire process with all partners; and in this way, all partners must be able to identify the benefits that the research can bring to them, which can range from publications to lobbying or community-based activities. The entire research process should be based on a survivor-centered approach, which prioritizes survivors’ safety and participation and makes sure the research does not harm. Such an approach should be responsive to the local context; be based on the building of equal relationships between HIC and LMIC researchers and participants; allow space to reflect on and adapt to the different perspectives and positions of all partners, and in this way explicitly aim to bring about change that is meaningful for the participants. Finally, the guidelines set out the practical steps to make sure that the research plan reflects these ethical and safety considerations. The ENGAGE guidelines can be downloaded for free by researchers from HICs and LMICs, and shared with participants so that they can be used as a basis for establishing research partnerships. The discussions among LMIC and HIC researchers and participants based on these guidelines should result in a statement of intent about the research, signed by all those involved in it, including the participants. Such a statement should clarify everyone's role in the research; agree on outcomes, methods, and timeframes; set adequate expectations; reflect relationships of equality and transparency in the research process; and create a system of accountability. The ENGAGE guidelines are intended to make participants more aware of their rights and the possibility to refuse research participation, and researchers of their responsibility to prevent research duplication and re-traumatization. In this way, they aim to promote research that is meaningful for survivors in terms of topics and outputs and to overcome many of the ethical risks described in this article.

## Conclusion

This article has described some of the ethical and practical challenges, and also some good practices, identified by Kenyan researchers and civil society representatives involved in different ways with international GBV research. Many of these challenges are related to the specific inequalities produced by cross-national research, including economic and epistemic inequalities, and imbalances in terms of risk and reward. These challenges can make international GBV research an exploitative experience for both participants and researchers from LMICs. These power inequalities compound the already existing, and well-documented challenges inherent in GBV research more generally. The newly developed ENGAGE guidelines complement existing guidelines on GBV research by focusing particularly on cross-national participatory research projects involving researchers or funders from HICs and partners and participants from LMICs. The guidelines thus serve as an instrument and tool kit for all those involved in international GBV research, to make sure that research with GBV survivors ceases to be an exploitative experience, and instead contributes to the transformation of survivors’ lives and the elimination of GBV around the world.
